# Synthesis and Study of the Optical Properties of a Conjugated Polymer with Configurational Isomerism for Optoelectronics

**DOI:** 10.3390/ma16072908

**Published:** 2023-04-06

**Authors:** Oscar Javier Hernández-Ortiz, Damaris Castro-Monter, Ventura Rodríguez Lugo, Ivana Moggio, Eduardo Arias, María Isabel Reyes-Valderrama, María Aurora Veloz-Rodríguez, Rosa Angeles Vázquez-García

**Affiliations:** 1 Área Académica de Ciencias de la Tierra y Materiales, Universidad Autónoma del Estado de Hidalgo, Carretera Pachuca-Tulancingo Km. 4.5, Ciudad del Conocimiento, Mineral de la Reforma 42184, Hidalgo, Mexico; 2 Laboratorio de Química Supramolecular y Nanociencias de la Unidad Profesional Interdisciplinaria de Biotecnología del Instituto Politécnico Nacional, Av. Acueducto s/n Barrio la Laguna Ticomán, Ciudad de México 07340, Ciudad de México, Mexico; 3 Centro de Investigación en Química Aplicada, Enrique Reyna H. 140, San José de los Cerritos, Saltillo 25294, Coahuila, Mexico

**Keywords:** optoelectronics, photochromic polymer, configurational isomerism, optical properties

## Abstract

A π-conjugated polymer (PBQT) containing bis-(2-ethylhexyloxy)-benzo [1,2-b’] bithiophene (BDT) units alternated with a quinoline-vinylene trimer was obtained by the Stille reaction. The chemical structure of the polymer was verified by nuclear magnetic resonance (^1^H NMR), Fourier transform infrared (FT-IR), and mass spectroscopy (MALDI-TOF). The intrinsic photophysical properties of the solution were evaluated by absorption and (static and dynamic) fluorescence. The polymer PBQT exhibits photochromism with a change in absorption from blue (449 nm) to burgundy (545 nm) and a change in fluorescence emission from green (513 nm) to orange (605 nm) due to conformational photoisomerization from the *trans* to the *cis* isomer, which was supported by theoretical calculations DFT and TD-DFT. This optical response can be used in optical sensors, security elements, or optical switches. Furthermore, the polymer forms spin-coated films with absorption properties that cover the entire visible range, with a maximum near the solar emission maximum. The frontier molecular orbitals, HOMO and LUMO, were calculated by cyclic voltammetry, and values of −5.29 eV and −3.69, respectively, and a bandgap of 1.6 eV were obtained, making this material a semiconductor with a good energetic match. These properties could suggest its use in photovoltaic applications.

## 1. Introduction

In recent decades, interest in the development and research of organic semiconductor materials, such as small molecules [1,2] and conjugated polymers [3,4] has gained great interest in the scientific community [5,6,7]. This is due to the attractive photophysical and processing properties of this type of material, which have been tuned and adapted for their application in optoelectronic devices such as organic light-emitting diodes (OLEDs) [8,9], organic solar cells (OSC) [1,10], organic field-effect transistors OFETs [11,12], perovskite solar cells [13,14], sensors [15,16,17], nonlinear optics materials [18,19], molecular switches [20], optical data storage [21], and others. The optical and electrical properties of organic semiconductors derive from their chemical structure [6], and thanks to the versatility of organic chemical synthesis, it is possible to tune them by molecular design [22], using different acceptor or donor segments to promote and favor intramolecular charge transfer (ICT) [23]. ICT denotes the charge transfer between donor-acceptor (D-A) groups located within a molecule during the excited state [24], which increases the electric transition dipole moments between the ground and excited states [25]. Intramolecular charge transfer processes result in bandgap (Eg) reduction [26], which is an important property for applications in solar cells [27], but also in other emerging applications such as environmental ones [28]. The optimal values are between 1.0 and 1.7 eV [26]. Although in the case of polymers, it depends on the reference point that is taken [29], low bandgap polymers are considered those with Eg less than 1.6 eV [30].

Conjugated polymers are particularly advantageous because of their processability, which enables the fabrication of high-performance, large-area devices [7]. Quinoline, a weakly basic compound, is one of the most interesting compounds for the development of organic materials for optoelectronics [31]. Quinoline and its derivatives [32,33,34] exhibit high thermal and oxidative stability, good processability in films, high photoluminescence performance (PL), and excellent electron transport properties [35]. Quinoline-based polymers with photochromic properties have been reported [36]. In these materials, quinoline enables modulation of the optical and electronic properties of the polymers [37], which have been shown to be excellent electron acceptors and transport materials [38,39,40]. Another interesting unit is benzodithiophene (BDT); this moiety has a rigid fused ring structure that makes it an electron-rich donor building block [41]. Therefore, benzodithiophene and its derivatives [42,43,44,45] have planar conjugated structures, tight and regular stacking, and excellent carrier transfer properties [46,47]. Optoelectronic devices based on BDT polymer donors often exhibit high performance, especially in photovoltaic devices [41,42,48,49].

Modulation of the optoelectronic properties of conjugated polymers by external stimuli such as light, heat, pH, and mechanical forces is a challenge for modern organic electronics [50,51]. Photochromic molecules are attractive, dynamically responsive systems for the fabrication of smart materials used in molecular switches and advanced photonic devices such as photodetectors and organic field-effect transistors (OFETs) [20,51,52]. Photochromic molecules can exhibit reversible photoinduced transformations between two photoswitchable states [53,54]. Therefore, photochromic molecules can be based on reversible [55], *trans/cis* [56,57] photoisomerization, or ring-opening/ring-closing photocyclization reactions [20,53]. Reversible switching between these binary states can be brought about by illumination with UV-visible light, which involves reversible changes in physical properties such as π-conjugation, dipole moment, ionic state, steric conformation, optical absorption, emission, the energy level of the highest occupied molecular orbital (HOMO) and the lowest unoccupied molecular orbital (LUMO), and others [20,54]. For photoisomerization to occur, there must be enough free space for the fragment to freely rotate [55,56]. The molecular design allows the photoisomerization behavior of a polymer to be modulated and controlled, ensuring a tunable photoreaction [52]. Photochromic polymers are often designed to promote a conformational change in the pendant groups of the polymer [50,58]. Most of the reported molecules with molecular transformation (*trans → cis → trans*) are based on an azo fragment [36,58,59,60], but this is not the only option [61].

In this work, we report the synthesis of a BDT-alternated quinoline-vinylene polymer that exhibits photochromism due to photoisomerization, causing a conformal change from trans to *cis* of the conjugated backbone. Quinoline and benzodithiophene segments were used for the design and synthesis of the polymer. The obtained results were supported by theoretical studies that helped to explain the change in optical properties caused by conformational isomerism.

## 2. Materials and Methods

The solvents used in the synthesis were distilled from desiccants before use. All chemicals were commercially available under the Sigma-Aldrich brand (St. Louis, MO, USA).

### 2.1. Synthesis

#### 2.1.1. 1,4- bis (octyloxy)-2,5-bis [(4-(6-bromoquinolin-2-vinyl)-yl)] benzene (**3**)

It was synthesized according to the reference [9].

#### 2.1.2. Polymer **PBQT**

Into a two-necked round-bottomed flask containing 129 mg (0.162 mmol) of **3**, 105 mg (0.135 mmol) of 2,6-bis(trimethyl tin)-4,8-bis(2-ethylhexyl)benzo[1,2-b:4,5b′]bithiophene (BDT), and 10 mg (0.015 mmol) of tetrakis(triphenylphosphine)palladium (Pd (0)) were added under nitrogen to 5 mL anhydrous toluene via cannula. The flask was subjected to three cycles of vacuum/N_2_ and allowed to react under stirring at 90 °C for two days. After cooling, 5 mL of distilled water was added, and the organic phase was extracted with CH_2_Cl_2_. The solvent was evaporated to its minimum volume, precipitated in methanol, centrifuged, and dried in a vacuum oven at 80 °C for 3 h. A violet solid with a melting point of 230 °C was obtained. ^1^H NMR (400 MHz, CDCl_3_, δ ppm): 8.86–6.93 (aromatic protons), 8.11 (d, J = 16), 7.14 (d, J = 16), 4.79 (protons bridging oxygen), 3.51 (α protons to oxygen), 1.63 (aliphatic protons), and 1.28 (s, CH_3_). FT-IR (cm^−1^): 3051, 2917, 2850, 1732, 1592, 1492, 1311, 1191, 1010, 964, 823, 715, 542, 455. UV-Vis (nm): λ_max_ = 446.

### 2.2. Molecular Design and Theoretical Calculations

Density functional theory (DFT) calculations were performed using Gaussian 09w [62] at the B3LYP/6-31G (d) level of theory in vacuum. The calculations of the frontier molecular orbitals were performed with the optimized geometry. The linear transitions of the absorption were estimated from the optimized molecular structures using TD-DFT at the BH and HLYP/6-31G (d, p) level of theory in vacuum.

### 2.3. Characterization

Structural characterization of the synthesized compounds was performed using a Bruker Ascend, an Oxford Varian, and a Bruker Biospin nuclear magnetic resonance spectrometer (Billerica, MA, USA), all operating at 400 Hz and analyzing the ^1^H nucleus. A PerkinElmer Spectrum GX Fourier transform infrared spectrophotometer (FT-IR) was used in transmission mode (Waltham, MA, USA), on powder samples, and at room temperature. The photophysical properties were determined in HPLC-grade chloroform. UV-vis absorption was performed using a PerkinElmer LAMBDA XLS spectrophotometer. Fluorescence spectroscopy was performed using a PerkinElmer LS55 and a Horiba PTI Quantamaster QM-8450-22-c spectrofluorimeter. In the latter case, the slits were fixed in such an order that the uncorrected spectra remained below the linear detection range (10^6^ counts). The fluorescence quantum yield was calculated from the emission spectra obtained with the integrating sphere (K petite sphere) of the same instrument. The excitation wavelength was set at 10 nm below the main absorption peak. At least four solutions with an absorbance value at the excitation wavelength of less than 0.1 were analyzed. The following optical properties were calculated from the spectra: (1) the half-height bandwidth (HHBW, nm) of absorption and emission as the distance in nanometers at 0.5 intensity in the normalized spectra; (2) the Stokes shift (cm^−1^) as the difference between the emission maximum and the absorption maximum of the normalized spectra, both first converted to cm^−1^; (3) the energy of the first excited state (E_1.0_, eV) as the point where the normalized absorption and emission spectra intersect; (4) the optical bandgap (Egopt, eV) was calculated from the optical absorption set point, following the methodology applied to π-conjugated systems [63]. A Bruker Autoflex Max mass spectrometer MALDI-TOF was used to determine the molecular weight of the macromolecules. Cyclic voltammetry (CV) measurements were performed on a PARSTAT^®^ 2273 electrochemical instrument at room temperature under a nitrogen atmosphere with a scan rate of 50 mV/s serving as the reference electrode, with a 3-electrode cell (an Ag/AgCl reference electrode, a Pt filament as a counter electrode, and as the working electrode, ITO covered with the film of the compound) in a solution of 0.1 M tetrabutylammonium hexafluorophosphate (Bu_4_NPF_6_) in anhydrous acetonitrile.

## 3. Results

### 3.1. Synthesis

**Scheme 1:**
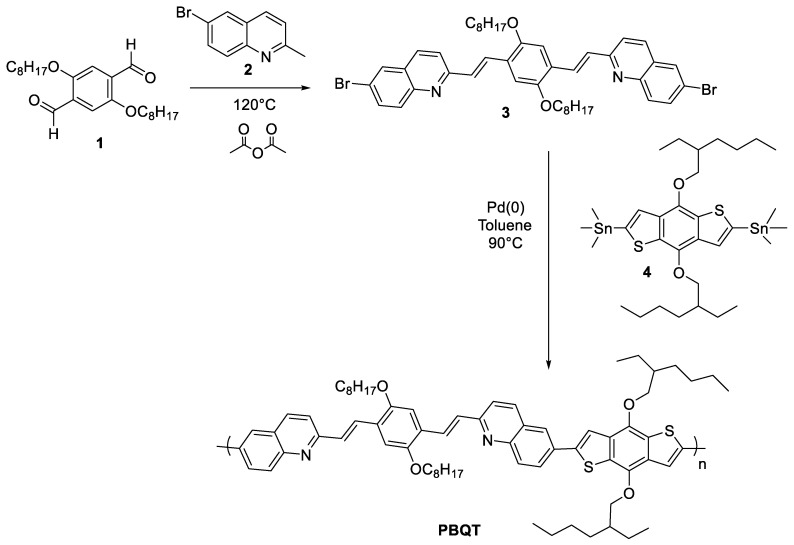
Reaction route to obtain PBQT. Compounds 1 (2,5-bis(octyloxy)terephthalaldehyde) and 2 (6-bromo-2-methylquinoline) are the reagents to obtain compound 3 by a Knoevenagel condensation. The Stille coupling is carried out between compounds 3 and 4 to obtain the PBQT copolymer.

**Figure 1:**
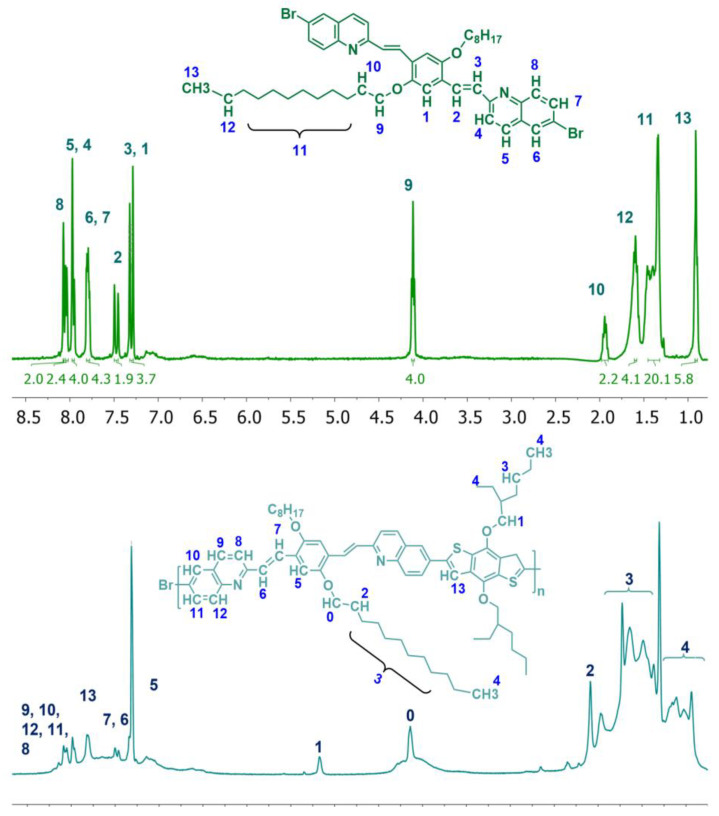
Comparative ^1^H NMR spectra of trimer **3** and **PBQT** using CDCl_3_ as solvent.

The chemical route for the synthesis of the polymer (**PBQT**) is shown in Scheme 1. The synthesized compounds were characterized by ^1^H NMR and FT-IR. Figure 1 shows the NMR spectra of **3** and **PBQT**. The ^1^H NMR spectrum of **3** shows the signals of quinoline protons ranging from 8.08 to 7.68 ppm, along with those of phenyl protons in 2,5-bisoctyloxyvinylidene. The vinyl protons are centered at 7.48 and 7.31 ppm, both of which are doublets integrating for two protons with coupling J constants of 16 Hz, indicating an *E* isomer. The protons from the methylene alpha of the alkoxy are shown at 4.11 ppm as triplets integrating to four protons. The rest of the methylenes of the aliphatic chains are shown in the range from 1.94 to 1.33 ppm as three signals integrating to 28 protons, while the triplet at 0.90 ppm is of the methyl. The proton signals of the polymer **PBQT** are broader than those of the corresponding monomers. It was observed that the signal of the trimethylstannyl protons centered at 0.45 ppm disappeared and, in contrast, the resonant protons could be identified near the bromine, indicating that all the chains are bromine terminated. Two other characteristic signals of the BDT that are visible are the alpha protons of the ethylhexyl chains at 4.23 ppm (2) and the one at 1.88 ppm (5).

**Figure 2:**
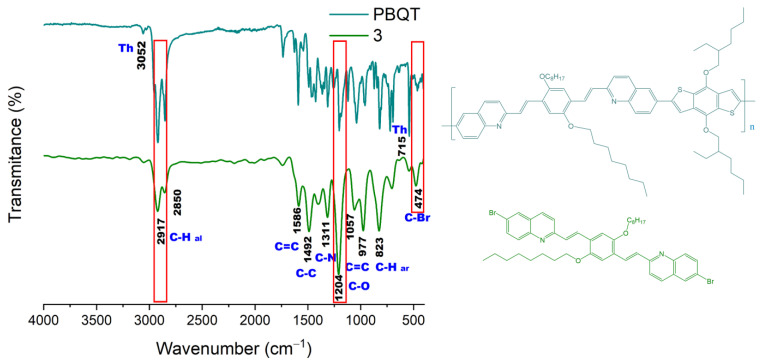
Comparative FTIR spectra of compound **3** and **PBQT** in thin film from CHCl_3_ solutions.

The FTIR spectra of trimer **3** and **PBQT** are shown in Figure 2. In general, both compounds show common signals: the vibrational modes of the C-O-C bond are at 1204 cm^−1^ and 1057 cm^−1^, while those of the vinyl bond C=C appear at 1586 cm^−1^, and the characteristic band of the disubstituted *trans* bond is found at 977 cm^−1^. The vibration of C-H out of the plane of the aromatic ring is at 823 cm^−1^, while that of the C-C bond is at 1492 cm^−1^. In addition, the vibrational modes of the C-H bond of the aliphatic chains are found at 2917 and 2850 cm^−1^. The band at 474 cm^−1^ is assigned to the C-Br vibration of the quinolines in trimer **3**, while the low intensity of this band in the spectrum of **PBQT** confirms that polymer chains are bromine terminated. The band observed at 715 cm^−1^ in the IR spectrum of **PBQT** corresponds to the C-S bond of thiophene, which is not present in trimer **3**, from which it is derived. The FTIR spectrum of **PBQT** FTIR (Figure 2) also shows the vibration of the C-N bond of the heteroatom at 1317 cm^−1^ and that of the C-S bond of the thiophene at 715 cm^−1^. The calculated average molecular weight (Mw) of **PBQT**, number average molecular mass (Mn), and polydispersity index (PI) using PS standards and the refraction index as detector are Mw = 4.466 Da, Mn = 2284 Da, and PI = 1.95.

### 3.2. DFT Study

**Figure 3:**
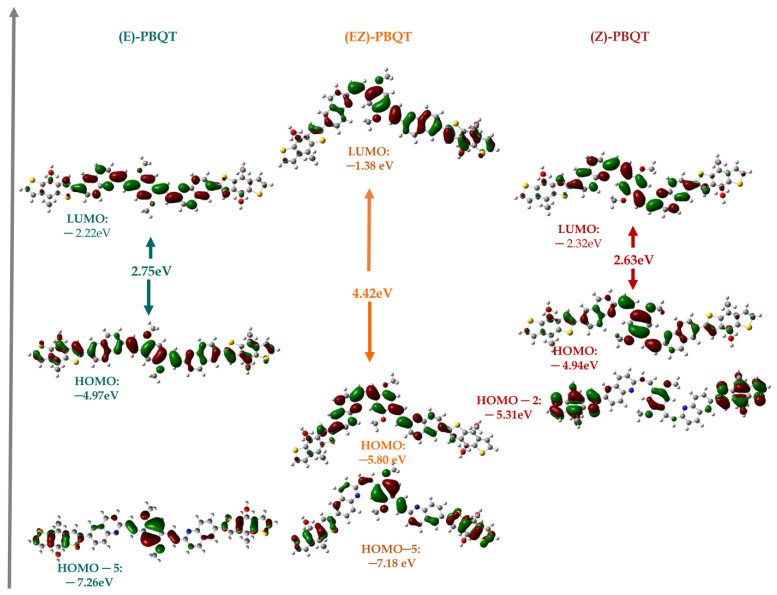
Isoimages of the molecular orbitals of **PBQT**, lateral chains were omitted for computational purposes.

The HOMO and LUMO energy levels for **PBQT** were calculated using the B3LYP/6-31G+ (d, p) level, and the visual representations of the HOMO and LUMO orbitals were calculated to visualize the electron density distribution as shown in Figure 3. Table 1 shows the vertical excitation energy Etr (eV), the theoretical absorption λ_max_(nm), the oscillator strength (O.S, ƒ), and the molecular orbital character (MO/character) together with the main excitation configuration for the repeating unit of **(E)-PBQT**, **(EZ)-PBQT**, and **(Z)-PBQT** polymer conformations. These values were calculated using the method TD-DFT/BhandHLYP/6-31 G+ (d, p) from the optimized structures obtained at the B3LYP/6-31G+ (d, p) level. The obtained bands for the electronic transitions show that the lowest electronic singlet excitation is characterized as a typical π-π*-transition. Note that when *cis* and *trans* conformations are combined, the structure is distorted, affecting the electronic delocalization of the repeating unit. This would not only have a negative effect on the optical and electronic properties, but it would also be less stable.

**Figure 4:**
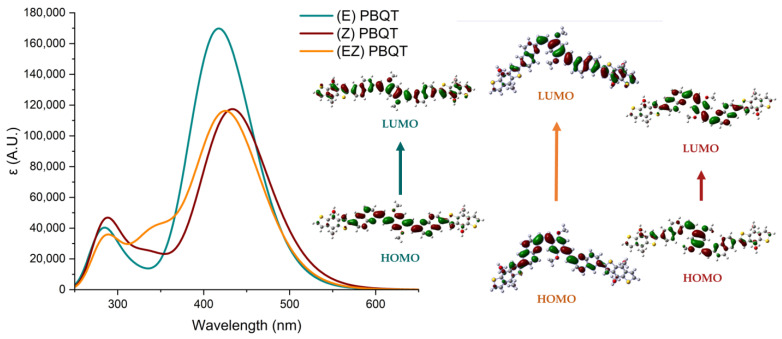
TDDFT absorption spectra of polymer **PBQT** obtained by applying BHandHLYP/6-31 (d, p) functional. At the right, Frontier molecular orbitals of **PBQT** in its *cis* (**(Z) PBQT**), *trans* (**(Z)-PBQT**), and intermediate (**(EZ) PBQT**) conformations.

Figure 4 shows the absorption spectra obtained from the TD-DFT calculation for the repeating unit of **(E)-PBQT**, **(EZ)-PBQT**, and **(Z)-PBQT** polymer conformations. These spectra show two absorption bands, the first at 280 nm and the second at 417 nm for **(E)-PBQT**, 426 nm for **(EZ)-PBQT**, and 433 nm for **(Z)-PBQT**. It can be observed that the theoretical and experimental spectra coincide at the main electronic transition. According to Table 1, the predominant transition is between the HOMO–LUMO orbitals. The first absorption band is attributed to a transition between HOMO-5 and LUMO for **(E)-PBQT** and **(EZ)-PBQT**, according to the TD-DFT study, and between HOMO-2 and LUMO energy levels for (**Z)-PBQT**. The second band is attributed to the transition between HOMO and LUMO energy levels. In the theoretical spectra, a red shift is observed when a conformational change from *trans* to *cis* occurs. This suggests that in the case of conformational isomerism caused by light exposure, there would be a shift in the absorption spectrum towards red. It should be noted that this shift may be even more pronounced in the presence of intermolecular interactions. This behavior is consistent with the experimental spectra (see further). Table 1 shows the estimated electronic transitions for **PBQT**; the total of the molecular orbital characters and the absorption spectra obtained from the TD-DFT calculation for trimer **3** can be found in the Supplementary Material.

### 3.3. Optical Properties

**Figure 5:**
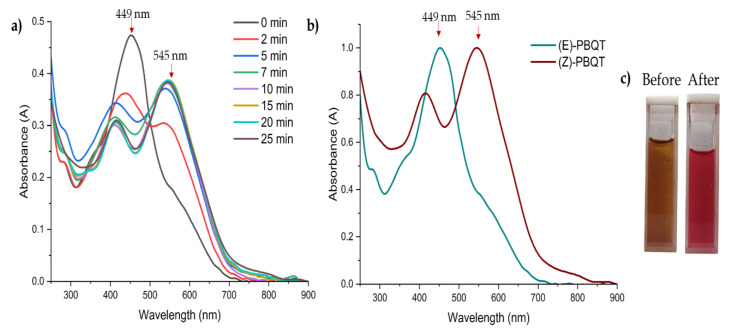
(**a**) Absorption spectra of **PBQT** in chloroform at different irradiation times; as the irradiation time increases, a bathochromic shift in the absorption of **PBQT** is observed. (**b**) Absorption spectra of **PBQT** in chloroform before and after irradiation; (**c**) **PBQT** solutions before being irradiated and in their final state after irradiation, an evident colorimetric change can be observed.

Figure 5a shows the evolution of the absorption spectrum of **PBQT** in chloroform when irradiated with white light from the LED. At time 0, the absorption spectrum presents a maximum absorption wavelength of 449 nm due to the extended conjugation (HOMO-LUMO electronic transition). Furthermore, a shoulder appears at 542 nm, which can be attributed to intramolecular charge transfer—as reported for donor-acceptor polymers [64]. However, along with the irradiation, this latter increases in intensity, converting to the main peak after 10 min, the time in which the optical change is practically complete. This indicates photochromism, and this type of response is attributed to diastereoselective photochromism, i.e., a photoinduced change in the absorption spectrum between two diastereoisomers due to *cis* and *trans* conformations in their molecular structure. This indicates photochromism, and this type of response is attributed to diastereoselective photochromism, i.e., a photoinduced change in the absorption spectrum between two dia-stereoisomers due to cis and trans conformations in their molecular structure. In agreement with the theoretical studies, the spectrum before irradiation is attributed to (E)-PBQT and after irradiation to (Z)-PBQT, as shown in Figure 5b. Theoretical calculations also suggest a distortion in the backbone that affects the electronic delocalization when there is a mixture of the two conformations, which explains the presence of both peaks in the intermediate times. Therefore, it is proposed that the most stable states are those in which the bulk of the polymer chain is accommodated in one conformation, either the *cis* or the *trans*. Accordingly, the optical bandgap Eg, which always has a value in the semiconductor range, decreases from **(*E*)-PBQT** to **(Z)-PBQT**. Photochromism can be used to apply these compounds in the manufacture of sensors, security tags and optical switches, as well as other advancing optoelectronic devices.

**Figure 6:**
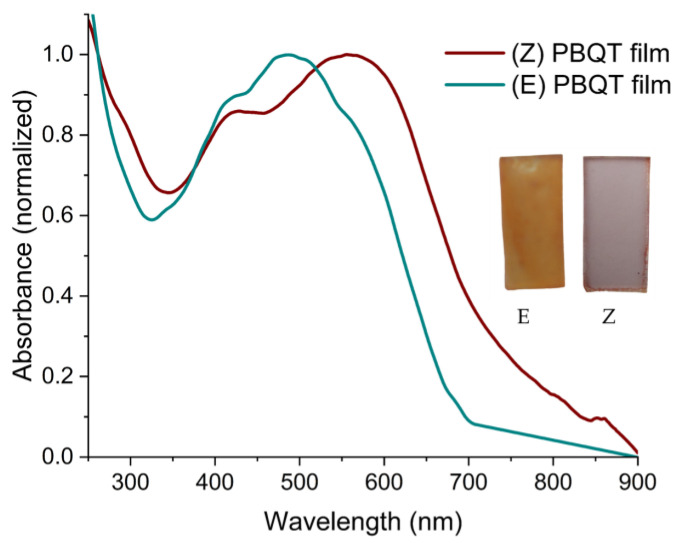
Normalized absorption spectra of **PBQT** in film.

Both isomers of the polymer could be deposited in thin films by spin coating. Figure 6 shows the corresponding absorption spectra. In both cases, the spectra cover most of the visible range, with the absorption maximum at 482 nm for the *trans*-form and at 545 nm for the *cis*-form, maintaining the same trend observed in the solutions and with a red shift due to solid state effects.

Table 2 summarizes the absorption properties of the molecules in chloroform and in film for both isomers. The small optical band gap (E_gopt_~1.6 eV), the broad absorption spectrum with large values of the half-height bandwidth (HHBWabs~500 nm), and the position of the absorption maximum (λabs~500 nm) near the maximum emission wavelength of the solar spectrum in uniform films indicate that **PBQT** could also be an excellent candidate for application in organic solar cells as a donor material.

**Figure 7:**
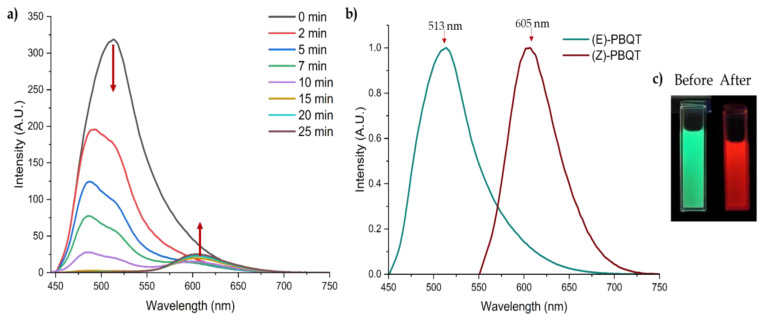
(**a**) **PBQT** emission spectra at different irradiation times, it is observed that when the solution is irradiated, the fluorescence intensity decreases and the red shifts. (**b**) Fluorescence spectra of **PBQT** in chloroform before and after irradiation; (**c**) **PBQT** solutions under the UV lamp before and after irradiation; note an evident change in the emission colour of **PBQT**.

Figure 7a shows the change in the emission spectrum at different times of irradiation. Just as with the absorption spectrum, there is a red shift in the spectrum when it is irradiated. The emission change is around 13 min. The total of the absorption and emission spectra taken at each minute is reported in the Supplementary Material. Figure 7b shows the comparison between the absorption and emission spectra in the chloroform solution of **PBQT** in the two possible conformations. It is evident that the bathochromic shift observed in the absorption is also present in the emission. **(E)-PBQT** shows narrower, stronger, and better-resolved emissions compared to **(Z)-PBQT**. In general, the polymer exhibits weaker emission compared to trimer **3** [9,65], which is likely due to intramolecular charge transfer attributed to the donor-acceptor character and this is enhanced in the Z conformation. As a consequence of the intramolecular charge transfer, the fluorescence spectra of both conformations are broad and not specular to their absorption counterparts. All fluorescence properties are summarized in Table 3. In resume, this polymer shows not only a colorimetric but also a fluorometric response. The change is reflected not only in the emission color, which turns from green to red, as shown in Figure 7c, but also in the fluorescence intensity, as evidenced by the 4.5-fold decrease in fluorescence quantum yield for the Z compared to the original E conformation.

### 3.4. Electrochemical Characterization

**Figure 8:**
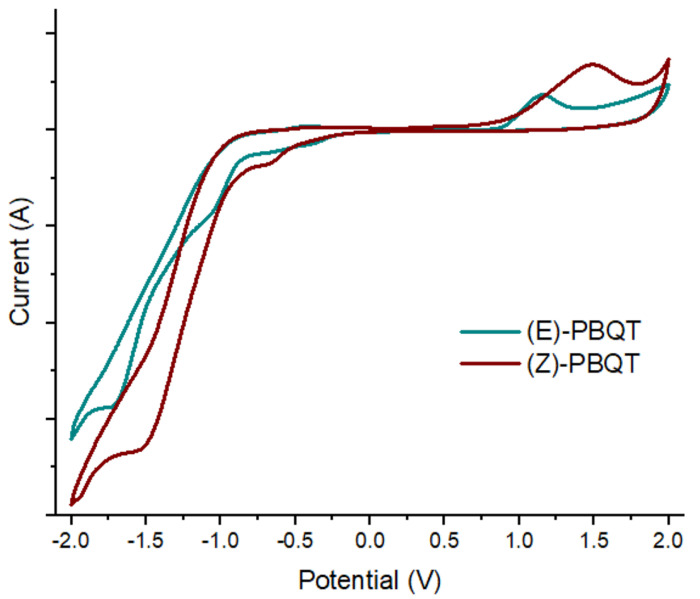
Voltammogram of **(E)-PBQT** in film vs. Ag/AgCl_2_, E_onset (Ox)_ = 0.89 V, E_onset (Red)_ = −0.77 V, Egap_E_ = 1.66 eV, and **(Z)-PBQT** in film vs. Ag/AgCl, E_onset (Ox)_ = 0.89 V, E_onset (Red)_ = −0.71 V, Egap_E_ = 1.60 Ev.

Cyclic voltammetry was used to evaluate the redox process on thin films deposited on ITO electrodes. The electrochemical data are summarized in Table 4. To obtain the values of the frontier orbitals (HOMO, LUMO), the oxidation and reduction potentials were determined at the onset (see Figure 8), which is defined as the potential at which the injection of holes or electrons into the HOMO or LUMO orbitals begins. An electrochemical band gap of 1.60 and 1.66 eV for **(E)-PBQT** and **(Z)-PBQT**, respectively.

**PBQT** is a polymer that exhibits photochromism due to a *trans→cis* isomerization in its backbone, which distinguishes it from other reports, as most macromolecules with photochromism attribute their response to transformations due to intramolecular reactions (cyclizations, ring openings, etc.) [66,67,68,69] and/or *trans→cis* transformations in the of the polymer’s pendant groups [50,58,70]. It also differs from some quinoline polymers with optical properties in that the quinoline segment is not located in the pendant group [36,71,72]. There is only one report of polymers with the quinoline sequenced in the backbone and with photoisomerism, but in that case, it causes a hypsochromic shift [73], whereas **PBQT** shows a bathochromic shift, which allows its possible use as a colorimetric or fluorimetric sensor. Its optical bandgap of 1.63 to 1.58 eV broadens the range of potential applications for this material, such as chemosensors [74], photoswitches [67], smart biomaterials [75], environmental applications [76,77], and optoelectronic devices in general [78,79]. In all these areas, both the absorption and emission properties of **PBQT** could have profited.

## 4. Conclusions

A semiconducting polymer bearing quinoline and bithiophene (BDT) units was synthesized by the Stille reaction and presents photochromism in both absorption and emission spectra. According to DFT and TD-DFT theoretical calculations, this is due to a conformational change from E to Z forms, i.e., a conformational change that occurs in the polymer backbone, not in the pendant groups as in most polymers described in the literature. Moreover, this behavior is seen in both solution and film, suggesting that the intramolecular spacing necessary for the transition between *trans* and *cis* is maintained. This makes **PBQT** a very interesting material to investigate for the fabrication of optical devices, in particular optical switches and colorimetric sensors. In addition to the photochromism that **PBQT** exhibits, it has a wide range of absorption covering from 400 to 800 nm, with a maximum at ≈500 nm that is close to the maximum solar emission, a low optical bandgap of ≈1.6 eV as found by UV-Vis spectroscopy, DFT, and cyclic voltammetry, and excellent film processing. Based on all of these properties, it is not excluded that it can also be used as an electron donor material in organic solar cells.

## Figures and Tables

**Table 1:** Electronic transition data obtained by the BhandHLYP/6-31G (d, p) method calculated for (**E)-PBQT**, **(EZ)-PBQT**, and **(Z)-PBQT**.

(E)-PBQT				(EZ)-PBQT				(Z)-PBQT			
λ_ab_ (nm)	E(tr) (eV)	OS (ƒ)	MO/Character	λ_ab_ (nm)	E(tr) (eV)	OS (ƒ)	MO/Character	λ_ab_ (nm)	E(tr) (eV)	OS (ƒ)	MO/Character
417.62	2.9688	3.7759	H→L (0.81)	426.33	2.9082	2.5552	H→L (0.81)	433.55	2.8597	2.6086	H→L (0.86)
325.3	3.8114	0.2114	H-2→L (0.48), H-1→L (0.25)	349.92	3.5432	0.6365	H-1→L (0.33), H→L + 1 (41%)	339.22	3.6549	0.4401	H-4→L (0.29), H-2→L (0.41)
297.19	4.1719	0.0222	H-3→L (0.34), H-1→L + 2 (0.12), H→L + 1 (0.13)	331.35	3.7418	0.2530	H-4→L (0.10), H-2→L (0.49), H-1→L + 1 (0.15)	318.7	3.8903	0.07	H-4→L (0.35), H-2→L (0.14), H-1→L + 1 (0.13)
282.4	4.3904	0.1939	H-3→L + 1 (0.13), H-2→L (0.13), H→L + 2 (0.18)	285.79	4.3382	0.3516	H-5→L (0.24), H-3→L + 1 (0.07) H-2→L (0.11), H-2→L + 2 (0.12), H-1→L + 3 (0.12)	287.95	4.3058	0.3897	H-5→L (0.23), H-1→L + 3 (0.16)
282.01	4.3965	0.577	H-5→L (0.27), H-2→L (0.17), H-1→L + 3 (0.14)	284.15	4.3633	0.2777	H-3→L + 1 (0.14) H-3→L (0.17), H-1→L + 1 (0.15), H→L + 2 (0.13)	285	4.3504	0.5477	H-2→L (0.21), H-1→L + 1 (0.15), H→L + 2 (0.16)

**Table 2:** Absorption optical properties in solution and film of the polymer **PBQT**.

Molecule		λ_abs_ Solution	HHBW_abs_ Solution	_Egopt_ Solution	ε (_λmax_)	λ_abs_ film	HHBWabs Film	E_gopt_ Film
		[nm]	[nm]	[eV]	ε [Lg^−1^ cm^−1^]	[nm]	[nm]	[eV]
PBQT	(E)-PBQT	350, 449 (max)	230	1.8	37.4	482	307	1.63
	(Z)-PBQT	400, 545 (max)	262	1.7	---	560	301	1.58

**Table 3:** Fluorescence optical properties in CHCl_3_ of the polymer PBQT.

Molecule	λem (λexc) [nm]	HHBWem [nm]	Δυ [cm^−1^]	E1,0 (λexc) [eV]	Φ (λexc) [%]	τ (λexc) [ns]	Krad 10^−11^ [s^−1^]	Knr 10^−11^ [s^−1^]
(E)-PBQT	513 (442)	134 (442)	2587	2.54 (442)	7.8 (442)	1.6 (455)	4.9	57.6
(Z)-PBQT	605 (538)	114 (538)	2893	2.05 (538)	1.7 (538)	0.5 (563)	3.4	196.7

**Table 4:** Electrochemical properties of **PBQT**.

Molecule	Onset Reduction (V)	Onset Oxidation (V)	IP HOMO (eV)	EA LUMO (eV)	Band Gap (eV)
(E)-PBQT	0.89	−0.77	−5.29	−3.63	1.66
(Z)-PBQT	0.89	−0.71	−5.29	−3.69	1.60

## Data Availability

Data is contained within the article or Supplementary Material.

## Supplementary Materials

The following supporting information can be downloaded at: https://www.mdpi.com/article/10.3390/ma16072908/s1.
