# Supplementary material for: Synthesis and Study of the Optical Properties of a Conjugated Polymer with Configurational Isomerism for Optoelectronics
Source: Materials (Basel). 2023 Apr 6;16(7):2908. doi: 10.3390/ma16072908 (PMC10096395; doi:10.3390/ma16072908)
Supplement: Supplementary file 1 [file materials-16-02908-s001.zip › materials-2209367-supplementary.pdf]

Supplementary material

Synthesis and Study of the Optical Properties of a Conjugated Polymer with Configurational Isomerism for Optoelectronics

Oscar Javier Hernández-Ortiz [1,2], Damaris Castro-Monter [1], Ventura Rodríguez Lugo [1,*], Ivana Moggio [3], Eduardo Arias [3], María Isabel Reyes-Valderrama [1], María Aurora Veloz-Rodríguez [1] and Rosa Angeles Vázquez-García [1,*]

[1] Área Académica de Ciencias de la Tierra y Materiales, Universidad Autónoma del Estado de Hidalgo, Carretera Pachuca-Tulancingo Km. 4.5, Ciudad del Conocimiento, Mineral de la Reforma 42184, Hidalgo, Mexico
[2] Laboratorio de Química Supramolecular y Nanociencias de la Unidad Profesional Interdisciplinaria de Biotecnología del Instituto Politécnico Nacional, Av. Acueducto s/n Barrio la Laguna Ticomán, Ciudad de México 07340, Ciudad de México, Mexico
[3] Centro de Investigación en Química Aplicada, Enrique Reyna H. 140, San José de los Cerritos, Saltillo 25294, Coahuila, Mexico
* Correspondence: venturar@uaeh.edu.mx (V.R.L.); rosavg@uaeh.edu.mx (R.A.V.-G.)

Contents

1. GPC results

2. TD-DFT data

3. Additional voltammograms

1. GPC Results of PBQT

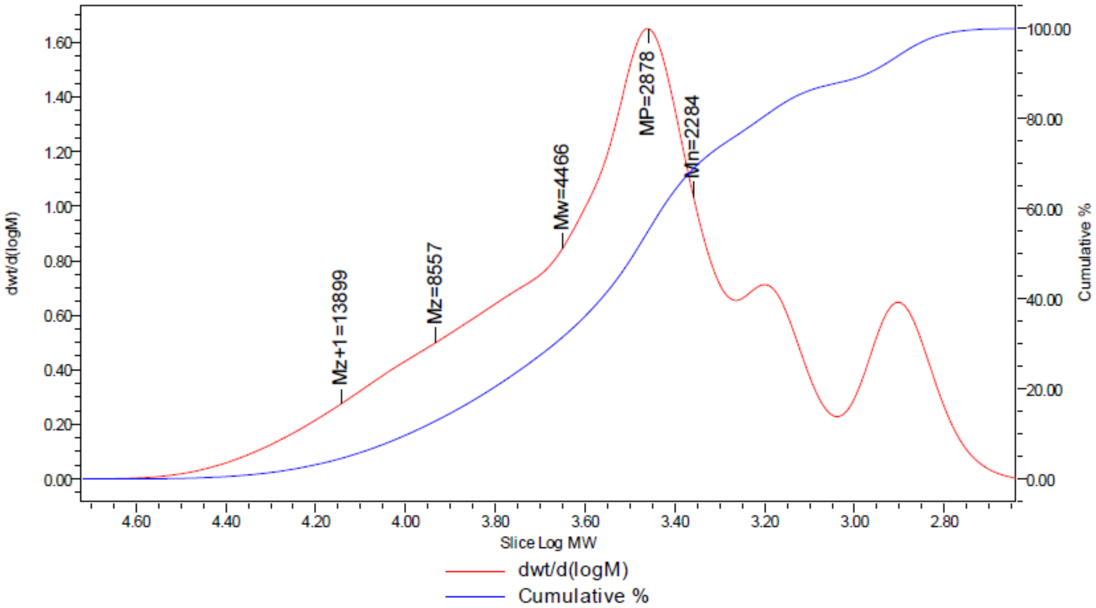

Figure S1. Gel permeation chromatography analysis of **PBQT** relative to polystyrene curves.

Table S1. GPC results

Retention time (min)	Mn (Dalton)	Mw (Dalton)	MP (Dalton)	Polydispersity
15.809	2,284	4,466	2,878	1.96

2. TD-DFT data

Table S2. Electronic transitions obtained by TD-DFT BhandHLYP/6-31 G (d, p) of trimer **3** Trans

**λ_{ab} (nm)**	**E(tr) (eV)**	***OS (f)***	***MO/Character***
402.19	3.0827	**2.1225**	H-1→L+1 (0.04), H→L (0.92)
322.92	3.8395	0	H-4→L (0.02), H-1→L (0.16), H-1→L+2 (0.05), H→L+1 (0.72)
305.71	4.0556	**0.1116**	H-3→L (0.03), H-2→L (0.73), H-1→L+1 (0.06), H→L+2 (0.06)
289.34	4.285	**0.1352**	H-5→L (0.06), H-4→L+1 (0.09), H-3→L (0.08), H-2→L (0.10), H-2→L+2 (0.04), H-1→L+1 (0.03), H-1→L+3 (0.11), H→L+2 (0.43)
287.88	4.3068	0	H-4→L (0.06), H-4→L+2(0.03), H-3→L+1(0.10), H-2→L+1(0.03), H-1→L (0.57), H-1→L+2(0.03), H→L+1(0.09), H→L+3(0.04),
276.33	4.4868	0	H-5→L+1(0.09), H-4→L (0.25), H-3→L+1(0.03), H-3→L+3(0.02), H-2→L+1(0.04), H-1→L (0.08), H-1→L+2(0.06), H-1→L+6(0.03), H→L+1(0.12) H→L+3(0.22)
270.71	4.58	**0.4229**	H-4→L+1(0.12), H-3→L (0.47), H-3→L+2(0.04), H-1→L+1(0.19), H→L+2(0.07)
252.97	4.9012	**0.1054**	H-6→L+1(0.03), H-5→L (0.20), H-4→L+1(0.03), H-4→L+3(0.03), H-3→L+2(0.02), H-2→L+2(0.05), H-1→L+1(0.13), H→L (0.03), H→L+2(0.32) H→L+14(0.05)
251.69	4.9262	0	H-6→L (0.04), H-4→L (0.28), H-3→L+1 (0.12), H-2→L+1 (0.10), H-1→L (0.03), H-1→L+2 (0.12), H→L+3 (0.22),
240.99	5.1448	0	H-6→L (0.02), H-4→L (0.04), H-4→L+2(0.02), H-3→L+3(0.05), H-2→L+1(0.33), H-1→L (0.06), H-1→L+2(0.04), H-1→L+6(0.02), H→L+3(0.35)

Table S3. Electronic transitions obtained by TD-DFT BhandHLYP/6-31 G (d, p) of trimer **3** Cis

**λ_{ab} (nm)**	**E(tr) (eV)**	***OS (f)***	***MO/Character***
422.3	2.9359	**1.4219**	H-2→L+1 (0.03), H→L (0.94)
332.47	3.7292	**0.1162**	H-2→L+1 (0.03), H-1→L (0.87)
330.82	3.7477	0	H-2→L (0.08), H-2→L+2 (0.03), H→L+1 (0.83)
295.25	4.1993	0	H-3→L+1 (0.05), H-2→L (0.71), H-1→L+1 (0.09), H→L+1 (0.09)
291.66	4.251	**0.0716**	H-5→L (0.04), H-4→L+1 (0.07), H-3→L (0.11), H-2→L+3 (0.10), H-1→L (0.03), H-1→L+2 (0.03), H→L+2 (0.55)
283.03	4.3806	0	H-5→L+1 (0.07), H-4→L (0.28), H-3→L+1 (0.06), H-3→L+3 (0.03), H-2→L+2 (0.09), H-2→L+4 (0.02), H-1→L+1 (0.07), H→L+3 (0.28)
272	4.5582	**0.3833**	H-4→L+1 (0.12), H-3→L (0.51), H-3→L+2 (0.04), H-2→L+1 (0.14), H→L+2 (0.11)
262.51	4.723	**0.0581**	H-6→L+1 (0.02), H-5→L (0.23), H-4→L+1 (0.04), H-4→L+3 (0.03), H-3→L+2 (0.04), H-2→L+1 (0.17), H-2→L+3 (0.02), H-1→L+2 (0.08) H→L (0.02), H→L+2 (0.23), H→L+12 (0.03)
257.1	4.8224	0	H-5→L+1 (0.04), H-4→L (0.25), H-3→L+1 (0.03), H-2→L (0.06), H-2→L+2 (0.03), H-1→L+1 (0.43), H→L+3 (0.08)
256.41	4.8353	0	H-4→L (0.1), H-3→L+1 (0.06), H-2→L (0.03), H-1→L+1 (0.29), H→L+3 (0.45)

Table S4. Electronic transitions obtained by TD-DFT BhandHLYP/6-31 G (d, p) of ***E*-PBQT**

λ_{ab} (nm)	E(tr) (eV)	*OS* *(f)*	*MO/Character*
417.62	2.9688	3.7759	H-4→L+1 (0.03), H-2→L (0.05), H-1→L+1 (0.06), H→L (0.81)
349.07	3.5519	0.007	H-3→L (0.06), H-2→L+1 (0.02), H-1→L (0.39), H-1→L+2 (0.05), H→L+1 (0.40)
325.3	3.8114	0.2114	H-3→L+1 (0.06), H-2→L (0.48), H-3→L+2 (0.03), H-2→L (0.25),H-3→L+3 (0.02), H-2→L (0.07)
312.18	3.9716	0.014	H-8→L (0.03), H-3→L (0.03), H-2→L+1 (0.24), H-1→L (0.21), H-1→L+2 (0.03), H→L+1 (0.23), H→L+3 (0.01)
300.59	4.1247	0.0215	H-5→L (0.2), H-5→L+2 (0.03), H-4→L (0.33), H-3→L+1 (0.09), H-2→L+2 (0.07), H-1→L+3 (0.06), H→L+4(0.07)
297.19	4.1719	0.0222	H-4→L+1 (0.1), H-3→L (0.34), H-3→L+2 (0.02), H-2→L+1 (0.03), H-2→L+3 (0.06), H-1→L (0.07), H-1→L+2 (0.12), H→L+1 (0.13)
293.98	4.2175	0.0909	H-8→L+1 (0.1), H-7→L (0.17), H-4→L (0.12), H-3→L+1 (0.03), H-3→L+3 (0.06), H-1→L+3 (0.03), H-1→L+5 (0.03), H→L+4 (0.24)
291.72	4.2501	0.0001	H-8→L (0.13), H-7→L+1 (0.07), H-6→L (0.04),H-4→L+3 (0.03), H-3→L+2 (0.03), H-3→L+4 (0.03), H-1→L(0.09), H-1→L+2(0.05), H-1→L+4(0.06), H→L+1(0.07), H→L+3(0.2), H→L+5(0.05)
282.4	4.3904	0.1939	H-5→L (0.12), H-4→L+2 (0.06), H-3→L+1 (0.13), H-2→L (0.13),H-1→L+1 (0.12), H→L (0.06), H→L+2 (0.18), H→L+4 (0.03)
282.01	4.3965	0.577	H-5→L (0.27), H-3→L+1 (0.03), H-2→L (0.17),H-2→L+2 (0.08),H-1→L+3 (0.14), H→L+2 (0.07)

Table S5. Electronic transitions obtained by TD-DFT BhandHLYP/6-31 G (d, p) of ***EZ*-PBQT**

λ_{ab} (nm)	E(tr) (eV)	*OS* *(f)*	*MO/Character*
426.33	2.9082	2.5552	H-2→L (2%)H-1→L(4%),H-1→L+1(3%)H→L(81%)
349.92	3.5432	0.6365	H-3→L (5%)H-2→L(3%),H-2→L+1(3%)H-1→L(33%)H-1→L+2(3%),H→L+1(41%)
331.35	3.7418	0.2530	H-5→L (2%)H-4→L(10%),H-3→L(3%)H-2→L(49%)H-2→L+1(2%),H-2→L+2(3%),H-1→L+1(15%)H→L+1(3%)H→L+2(4%)
317.15	3.9094	0.0237	H-4→L (3%)H-3→L+1(16%),H-1→L(23%)H-1→L+1(6%)H→L(2%),H→L+1(27%),H→L+3(7%)
310.23	3.9965	0.0007	H-5→L (19%), H-4→L(33%), H-3→L(4%), H-3→L+1(4%), H-2→L(6%), H-2→L+2(8%), H-1→L+1(4%), H-1→L+3(4%), H→L+4(3%)
299.82	4.1353	0.0407	H-4→L (7%)H-4→L+1(6%),H-3→L(29%)H-3→L+1(2%)H-2→L+1(5%),H-2→L+2(2%),H-2→L+3(4%), H-1→L(4%), H-1→L+2(10%), H-1→L+3(3%), H→L+1(8%)
294.41	4.2112	0.0507	H-8→L (11%)H-8→L+1(3%),H-7→L+1(4%)H-6→L(6%)H-6→L+1(3%),H-5→L(3%),H-4→L(7%), H-4→L+2(3%), H-3→L+3(4%), H-1→L+3(3%), H-1→L+5(2%), H→L+2(2%), H→L+3(3%), H→L+4(22%)
292.59	4.2374	0.0650	H-8→L (7%)H-7→L(6%),H-6→L(6%)H-6→L+1(6%)H-5→L+1(3%),H-3→L(3%),H-3→L+2(2%), H-3→L+4(4%), H-1→L(5%), H-1→L+2(3%), H-1→L+4(4%), H→L+1(3%), H→L+3(14%), H→L+4(6%), H→L+5(6%)
285.79	4.3382	0.3516	H-5→L (24%)H-4→L+2(2%),H-3→L+1(7%)H-2→L(11%)H-2→L+2(12%),H-1→L+3(12%),H→L+2(3%), H→L+4(4%)
284.15	4.3633	0.2777	H-5→L (5%)H-4→L+2(3%),H-3→L+1(14%)H-3→L(17%)H-1→L(2%),H-1→L+1(15%),H→L(5%), H→L+2(13%), H→L+3(5%), H→L+4(2%)

Table S6. Electronic transitions obtained by TD-DFT BhandHLYP/6-31 G (d, p) of ***Z*-PBQT**

**λ$_{ab}$ (nm)**	**E(tr) (eV)**	***OS (f)***	***MO/Character***
433.55	2.8597	2.6086	H-3→L+1 (0.02), H-2→L (0.02), H-1→L+1 (0.03), H→L (0.86)
352.47	3.5176	0.0076	H-3→L (0.06), H-2→L+1 (0.04), H-1→L (0.34), H-1→L+2 (0.03), H→L+1 (0.44)
339.22	3.6549	0.4401	H-5→L (0.04), H-4→L (0.29), H-2→L (0.41), H-1→L+1 (0.14), H→L+2 (0.02)
323.53	3.8323	0.0079	H-2→L+1 (0.21), H-1→L (0.29), H→L+1 (0.35), H→L+3 (0.07)
318.7	3.8903	0.07	H-5→L (0.13), H-4→L (0.35),H-3→L+1 (0.03), H-2→L (0.14), H-2→L+2 (0.07),H-1→L+1 (0.13), H-1→L+3(0.04), H→L+2 (0.02)
301.6	4.1109	0.0243	H-6→L (0.02), H-4→L+1 (0.10), H-3→L (0.38),H-2→L+1 (0.05),H-2→L+3 (0.07), H-1→L+2 (0.14), H→L+1 (0.07), H→L+3 (0.02)
294.84	4.2052	0.0632	H-9→L (0.02), H-8→L+1 (0.09), H-7→L (0.15),H-5→L (0.07),H-4→L (0.05), H-3→L+1 (0.03), H-3→L+3 (0.06), H-1→L+3 (0.02), H-1→L+5 (0.02), H→L+4 (0.26)
293.3	4.2272	0.0001	H-8→L (0.18), H-7→L+1 (0.07),H-5→L+1 (0.05),H-4→L+3 (0.03), H-3→L (0.05), H-3→L+2 (0.05), H-3→L+4(0.03), H-1→L(0.04), H-1→L+2(0.02), H-1→L+4(0.05), H→L+3(0.21), H→L+5(0.06)
287.95	4.3058	0.3897	H-5→L (0.23), H-4→L+2 (0.03), H-3→L+1 (0.09),H-2→L (0.09),H-2→L+2 (0.11), H-1→L+3 (0.16), H→L+2 (0.03), H→L+4 (0.05)
285	4.3504	0.5477	H-7→L (0.02), H-5→L (0.04), H-4→L+2 (0.03),H-3→L+1 (0.12),H-2→L (0.21), H-2→L+2 (0.03), H-1→L+1 (0.15), H→L (0.04), H→L+2 (0.16), H→L+4 (0.06)

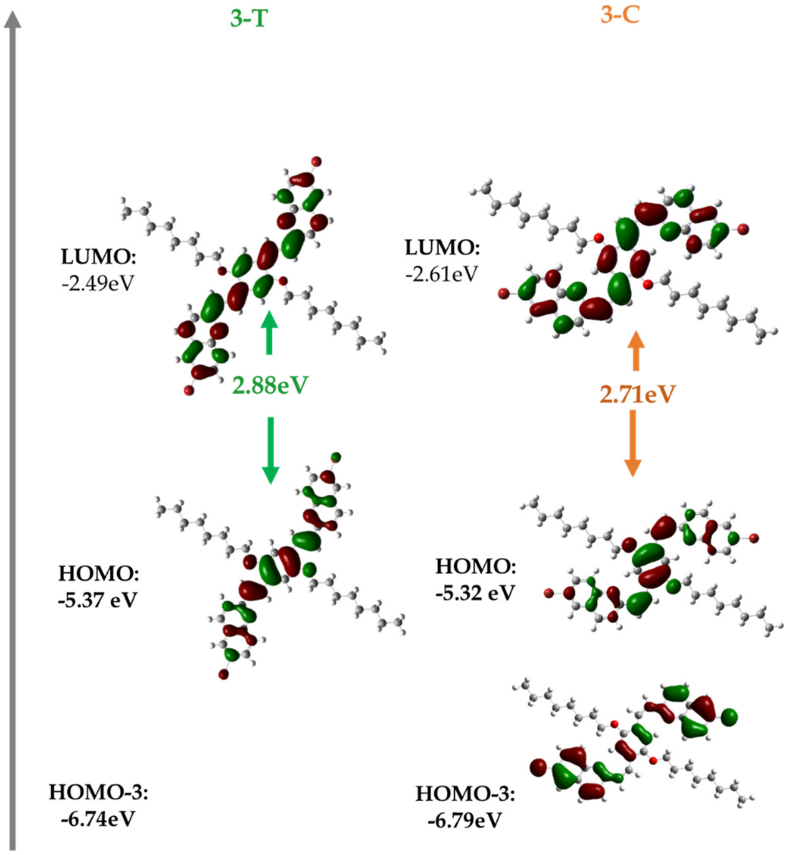

Figure S2. Isoimages of the molecular orbitals of trimer **3**.

Table S7. Electronic transition data obtained by the BhandHLYP/6-31G (d, p) calculation for **3T** and **3C**.

**3T**				**3C**			
**λab (nm)**	**E(tr) (eV)**	**OS (*f*)**	**MO/Character**	**λab (nm)**	**E(tr) (eV)**	**OS (*f*)**	**MO/Character**
402.19	3.0827	2.1225	H→L (0.92)	422.3	2.9359	1.4219	H→L (0.94)
305.71	4.0556	0.1116	H-2→L (0.73)	332.47	3.7292	0.1162	H-1→L (0.87)
289.34	4.285	0.1352	H-2→L+2 (0.04), H-1→L+3 (0.11), H→L+2 (0.43)	291.66	4.251	0.0716	H-3→L (0.11), H-2→L+3 (0.10) H→L+2 (0.55)
270.71	4.58	0.4229	H-4→L+1(0.12), H-3→L (0.47), H-1→L+1(0.19)	272	4.5582	0.3833	H-4→L+1 (0.12), H-3→L (0.51), H-2→L+1 (0.14),
252.97	4.9012	0.1054	H-5→L (0.20), H-1→L+1(0.13), H→L+2(0.32)	262.51	4.723	0.0581	H-5→L (0.23), H-2→L+1 (0.17), H→L+2 (0.23)

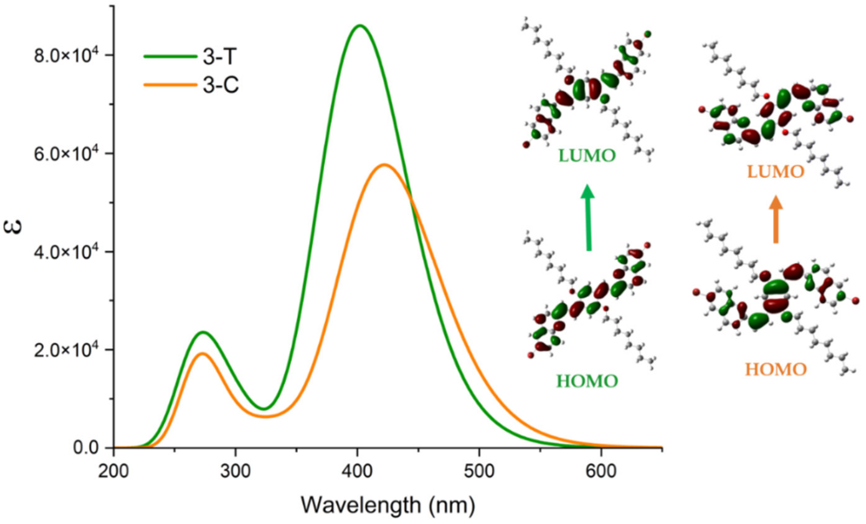

Figure S3. TDDFT absorption spectra of macromolecule **3** (3-C, 3-T) obtained by applying the BHandHLYP / 6-31 (d, p) methode. At right, Frontier molecular orbitals of **3** in its *cis* (3-C) and *trans* (3-T) conformation.

4. Additional voltammograms

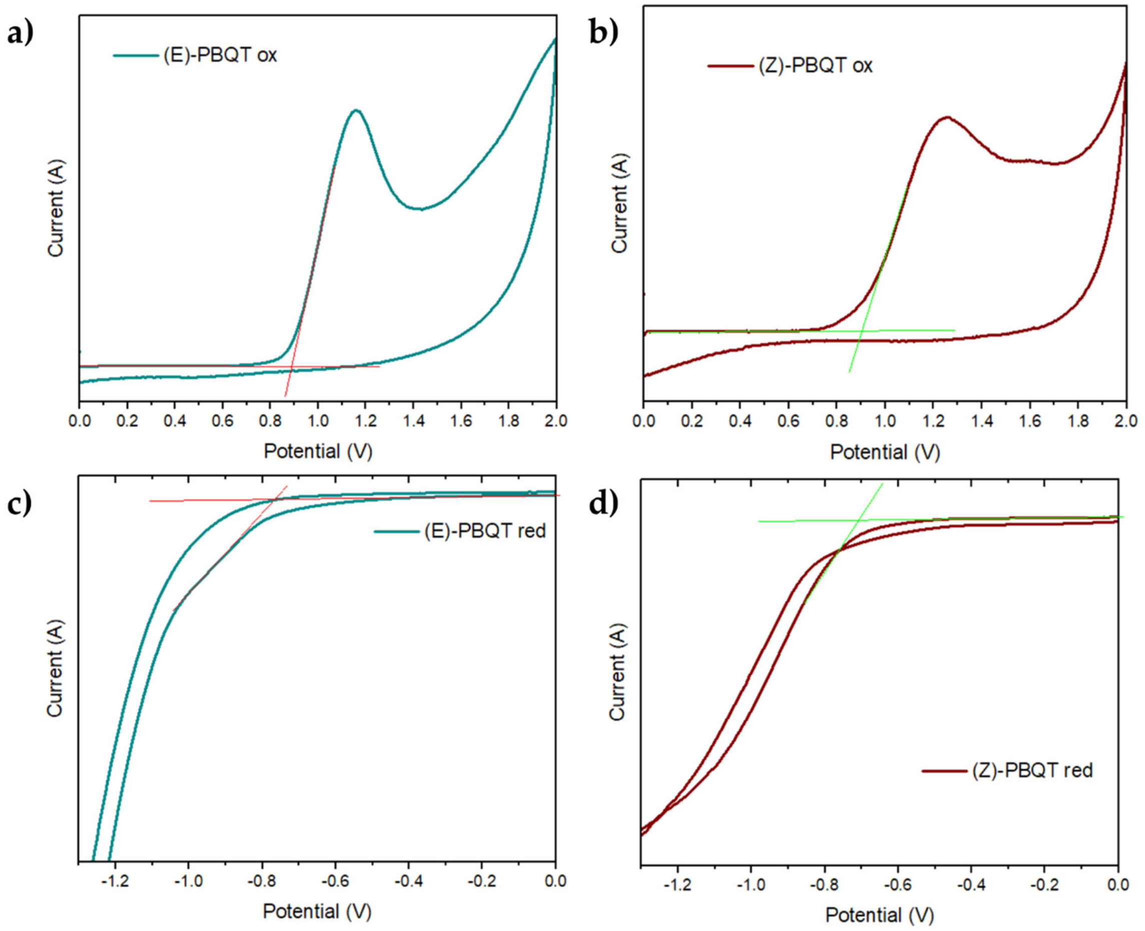

Figure S4. Voltammogram of **PBQT** vs Ag/AgCl. a) Cathodic region for ***E*-PBQT,** b) Cathodic region for ***Z*-PBQT,** c) anodic region for ***E*-PBQT** d) anodic region for ***Z*-PBQT.**
